# F56. IMPACT OF SIDE EFFECTS DUE TO SECOND-GENERATION ANTIPSYCHOTICS ON THE FUNCTIONING OF PATIENTS WITH SCHIZOPHRENIA: AN OBSERVATIONAL, PATIENT CENTERED, WEB SURVEY

**DOI:** 10.1093/schbul/sby017.587

**Published:** 2018-04-01

**Authors:** Rajiv Tandon, Catherine Weiss, William Lenderking, Owen Cooper, Huda Shalhoub, Leah Kleinman, Jun Chen, Ann Hartry, Mallik Green, Stine R Meehan, Laëtitia Bouérat Douvold

**Affiliations:** 1 University of Florida; 2 Otsuka Pharmaceutical Development & Commercialization, Inc; 3 Evidera; 4 Lundbeck LLC; 5 H. Lundbeck A/S

## Abstract

**Background:**

In patients with schizophrenia, antipsychotic medications, including second-generation antipsychotics, may cause many side-effects (SE) often leading to treatment discontinuation, and possible relapse as a consequence. The impact of treatments on patient-centered outcomes such as health-related quality of life (HRQOL) is less well understood. Even less well understood is the impact of side effects on patient-centered outcomes such as daily functioning and HRQOL. Therefore, the study’s primary goal was to gain a deeper understanding of the impacts of SEs of second generation antipsychotics on patients’ day to day functioning.

**Methods:**

A cross-sectional, web-based, patient-reported survey was fielded in the United States between July and November 2017. The final survey included patient socio-demographics, a quality of life measure (Quality of Life Enjoyment and Satisfaction Questionnaire Short Form, Q-LES-Q-SF), questions on treatment satisfaction, SEs experienced (Glasgow Antipsychotic Side-Effect Scale, GASS), and questions about the impact of SEs on functioning and emotions. Patients were recruited through patient advocacy and support groups, and medical research panels. Patient inclusion criteria: Self-reported schizophrenia diagnosis; 18 to 65 years old; stable for at least one month at time of screening; prescribed a second-generation antipsychotic medication for 1–12 months; the final sample consisted of those individuals who reported experiencing one or more side-effects based on the GASS.

**Results:**

The total sample (n=180) had a mean age of 35 (range 18–61) years old, of which 58.3% were females. Approximately a quarter (27.8%) of the sample had a college degree or higher; 69.4% identified as White, followed by 16.7% Black/ African American, and 6.1% Native Hawaiian/ Pacific Islanders. Most prevalent SEs reported on the GASS were ‘difficulty sleeping’ (81.1%), ‘feeling sleepy during the day’ (77.2%), ‘dry mouth’ (70.6%), and ‘feeling restless (60.6%). The SEs most commonly reported as distressing, for those patients experiencing that SE, were difficulty passing urine (23.3%), and feeling drugged/like a zombie (19.4%). The minimum impact from SEs on daily functioning was 53.2 on a 0–100 Visual Analogue Scale (higher number reflects more negative impact on daily functioning; 0=no impact and 100=very highly impacted). Across the SEs further probed about, the most severe impact was on one’s ‘ability to get or do a job’; specifically, for the SEs ‘shaky hands or arms’ the mean impact was 76.1, followed by 69.8 for restlessness. ‘Problems enjoying sex’ had the greatest effect on one’s ‘intimate relationships’ (mean 74.8), and feeling drugged/like a zombie had the greatest effect on one’s ‘ability to concentrate’ (mean 70.2).

**Discussion:**

The study indicates the importance of incorporating the patient with schizophrenia’s perspective when assessing SE experiences and impact on functioning due to second generation antipsychotic agents. Findings suggest that both activating SEs (restlessness) and sedating SEs (feeling drugged and sleepiness) have pronounced undesirable impact on daily patient functioning.

